# Discovery and Functional Characterization of SnFDHal, an Efficient Tryptophan 5-Halogenase from *Streptomyces noursei*

**DOI:** 10.1007/s12010-025-05449-0

**Published:** 2025-11-08

**Authors:** Hassan Sher, Haley A. Hardtke, Mark D. Gold, Sean J. Johnson, Y. Jessie Zhang, Jixun Zhan

**Affiliations:** 1 Department of Biological Engineering, Utah State University, Logan, UT 84322 USA; 2 Department of Molecular Biosciences, The University of Texas at Austin, Austin, TX 78712 USA; 3 Department of Chemistry and Biochemistry, Utah State University, Logan, UT 84322 USA

**Keywords:** *Streptomyces noursei*, Tryptophan 5-halogenase, Flavin-dependent halogenase, Thermostability, Structural modeling

## Abstract

Flavin-dependent halogenases (FDHs) are a class of enzymes renowned for their regioselective ability to precisely insert halogen atoms into small aromatic compounds. Halogen incorporation can enhance the physicochemical and biological properties of molecules, making them valuable for agrochemical and pharmaceutical applications. Through bioinformatic mining of bacterial genomes, we discovered and functionally characterized SnFDHal, an efficient tryptophan 5-halogenase from *Streptomyces noursei* NRRL B-1714. This halogenase operates across a broad pH range and exhibits a melting temperature of 46.7 °C at both pH 6 and pH 8, which is comparable to thermophilic halogenases such as Th-Hal and BorH, and notably higher than those of mesophilic counterparts. Steady-state kinetic analysis revealed that SnFDHal displays superior catalytic efficiency for the chlorination of L-tryptophan compared to other FDHs reported to date. Structural modeling of its active site suggests that a conserved bulky phenylalanine residue (F49) promotes halogenation at the C-5 position of L-tryptophan, consistent with experimental findings. The combination of high catalytic efficiency and thermostability positions SnFDHal as a promising biocatalyst for applications in agrochemical and pharmaceutical industries.

**Supplementary Information:**

The online version contains supplementary material available at 10.1007/s12010-025-05449-0.

## Introduction

Halogenation serves as a critical modification in the biotechnology and pharmaceutical sectors, improving the biological activity, physicochemical stability, and solubility of small molecules, such as therapeutic agents and agrochemicals [1, 2]. About 96% of agrochemical products, including fungicides, insecticides and herbicides, contain halogen atoms. More than 25% of the best-selling pharmaceutical drugs are halogenated and at least 63% of them require halogenation steps during the manufacturing process [3, 4]. Halogen substituents in antibiotic, anticancer and psychoactive compounds have profound effects on their biological properties such as lipophilicity, membrane permeability, and drug target interaction [5–8]. Currently, chemical halogenation is widely used for introducing halogen atoms into molecules. However, this method relies on harsh reaction conditions and hazardous halogen gases that often produce a mixture of products, making it difficult to selectively halogenate a target compound with precision [9, 10]. To circumvent the hazardous nature and lack of selectivity in the chemical halogenation process, bio-halogenation carried out by halogenases has become a promising alternative for synthetic applications because this greener approach uses enzymes with high regio- and stereo-selectivity [11–13].

Flavin-dependent halogenases (FDHs) are a class of enzymes especially valuable for their exceptional precision and regiospecificity in halogenating aromatic compounds [14, 15]. FDHs are a two-component halogenase/reductase system, in which the halogenase component uses FADH_2_ (reduced flavin adenine dinucleotide) that is generated by the reductase system (FRE) from FAD (flavin adenine dinucleotide), to produce hydroperoxy flavin [16]. This hydroperoxyl flavin then reacts with a halide ion to synthesize hypohalous acid (HOX). The HOX is then channeled approximately 10 Å to the distal substrate binding site through a dedicated tunnel. A conserved lysine located at the end of the tunnel plays a key role in activating HOX for accurate and efficient substrate electrophilic halogenation [17, 18].

FDHs are known for their regiospecific halogenation of aromatic small molecules such as tryptophan. In 2000, Keller et al. discovered the first flavin-dependent halogenase, PrnA, which catalyzes the halogenation of tryptophan at the C-7 position, the initial step in the biosynthetic pathway of the antibiotic pyrrolnitrin [19]. Since then, many other tryptophan halogenases with distinct regiospecificity have been identified. These include PyrH, AbeH, and *Xszen*FHal, which halogenate at the C-5 position [20, 21], SttH and ThHal, with C-6 specificity [22, 23], and KtzQ and RebH, which, like PrnA, target the C-7 position [19, 24].

In this study, we identified a new tryptophan 5-halogenase from the bacterial genome of *Streptomyces noursei* NRRL B-1714. *In vitro* characterization of this enzyme, named SnFDHal, demonstrated high efficiency and strict specificity for the synthesis of 5-chlorotryptophan. Molecular modeling of SnFDHal revealed that a bulky phenylalanine residue at position 49 (F49), previously implicated in halogenase regioselectivity, positions the tryptophan substrate to favor halogenation at the C-5 site while preventing modification at the C-6 or C-7 positions. This study reports the discovery of a novel tryptophan 5-halogenase, expanding the limited pool of characterized Trp-5-specific halogenases. The addition of SnFDHal enhances the growing repertoire of FDHs available for sustainable, regiospecific halogenation of small aromatic compounds in green chemistry applications.

## Materials and Methods

### General Equipment and Experimental Materials

*S. noursei* NRRL B-1714 was kindly provided by the Agricultural Research Service Culture Collection (NRRL - Northern Regional Research Laboratory) and re-cultured in Yeast extract/Malt extract (YM medium) at 28 °C with shaking (250 rpm) in preparation for genomic DNA extraction. Genomic DNA was extracted using the Quick-DNA™ Fungal/Bacteria DNA Miniprep Kit (Zymo Research, USA). Primers for amplification of the target halogenase gene were ordered from Thermo Scientific and dissolved in Tris–EDTA (TE) buffer to a final concentration of 100 ng/mL. The PCR reactions were conducted with an Arktik Thermal Cycler (Thermo Scientific). The T4 DNA ligase, Phusion High-Fidelity DNA polymerase, and restriction enzymes used in this study were purchased from New England Biolabs. *Escherichia coli* XL1-Blue and BL21 (DE3) were used for routine cloning and protein expression, respectively. Optical density measurements to estimate *E. coli* growth were taken using a Thermo Scientific GENESYS 20 UV-Visible Spectrophotometer. The halogenated samples were analyzed on an Agilent 1200 HPLC using an Agilent Eclipse Plus C18 reversed-phase analytical column (5 μm, 4.6 × 250 mm) at 230 nm. A SCIEX Triple Quad™ 7500 QTRAP^®^ Mass Spectrometer was used for determining molecular mass of the halogenated product. Bradford assay solution was purchased from TCI America (Portland, OR, USA). All other solvents and chemicals were purchased from Fisher Scientific.

### Bioinformatics Analysis and Strain Construction

The whole genome sequence of *S. noursei* NRRL B-1714 was obtained from the National Center for Biotechnology Information (NCBI) (https://www.ncbi.nlm.nih.gov/). The RAST annotation server was used to annotate the genome and reveal the presence of putative halogenase genes. A Basic Local Alignment Search Tool (BLAST) analysis was conducted on the annotated amino acid sequence of SnFDHal revealing significant similarities to various Trp halogenases. The amino acid and DNA sequences of this putative halogenase are presented in SI Table 1. Phusion high-fidelity DNA polymerase and a set of primers (SI Table 2) were used to clone the target gene from the genomic DNA of *S. noursei* NRRL B-1714. The PCR program used for amplification of the SnFDHal gene consisted of an initial denaturation at 98 °C for 5 min, 30 cycles of a regular program (98 °C for 30 s, annealing at 60 °C for 40 s, and extension at 72 °C for 90 s), followed by a final extension at 72 °C for 15 min. The PCR product of SnFDHal was ligated into the pJET1.2 vector to yield pJET-SnFDHal. The 1,551 bp gene insertion to pJET1.2 was confirmed by sequencing using the Sanger method at Eton Bioscience, Inc. The gene insert from pJET-SnFDHal was subsequently ligated into the pET28a(+) plasmid between the Nde1 and Xho1 sites to yield the expression plasmid pET28a-SnFDHal (SI Fig. 1A).

### Production and Purification of His_6_-tagged SnFDHal

The expression plasmid pET28a-SnFDHal was transferred into *E. coli* BL21(DE3) competent cells through chemical transformation. The colonies were picked from pET28a-SnFDHal/*E. coli* BL21(DE3) plates and grown in 5 mL of LB medium supplemented with kanamycin (50 µg/mL) and incubated at 37 °C with shaking at 250 rpm overnight. Then 4 mL of the seed culture was inoculated into 400 mL of LB broth with the same concentration of kanamycin. Cells were grown at 37 °C under the same conditions described above until the OD_600_ reached between 0.4 and 0.6, at which point gene expression was induced by adding 200 µM IPTG. The culture was maintained at 28 °C with shaking at 250 rpm for an additional 18 h. Cells were harvested by centrifugation at 4,000 rpm for 8 min, resuspended in the lysis buffer consisting of 20 mM Tris/HCl (pH 7.9) and 0.5 M NaCl, and disrupted by MISONIX sonication 3000 for 20 min (20 s on and 40 s off with an amplitude of 8.5) on ice. Cell debris was removed from the crude cell lysate by centrifugation at 13,000 rpm at 4 °C for 45 min. SnFDHal was purified by MCLAB nickel-nitrilotriacetic acid (Ni–NTA) agarose column chromatography using a buffer consisting of 50 mM monobasic phosphate and 10 mM NaCl, with various concentrations of imidazole. The purified His_6_-tagged protein was concentrated and desalted against 1× Native purification buffer (250 mM sodium phosphate, monobasic and 2.5 M NaCl) with centrifugal filter devices (30 K Millipore). The final purified protein was resolved on 12% SDS-PAGE and stained with Coomassie Blue (SI Fig. 1B).

### Halogenation Assays with SnFDHal

The flavin reductase (Fre) in *E. coli* BL21(DE3) was overexpressed and purified according to the previously reported protocol [25]. Each 100-µL halogenation reaction contained 9.6 mM NADH, 100 µM FAD, 10 mM NaCl, 1 mM substrate, 10 µM Fre, and 5.5 µM SnFDHal in 100 mM phosphate buffer (pH 6.0). The reaction mixture was incubated at 35 °C in a static incubator for 30 min. Reactions were quenched by 100 µL of methanol followed by centrifugation at 13,000 rpm for 10 min to remove precipitated proteins. Subsequently, the supernatants were analyzed by HPLC. A gradient elution was performed using solvent A (methanol) and solvent B (water containing 0.1% formic acid) under the following conditions: 0–35 min, A from 5% to 95%, at a flow rate of 1 mL/min. To investigate the reaction time course, additional reaction mixtures were incubated for up to 2 h, with samples quenched at designated time intervals and analyzed using the same HPLC method described above.

### Determination of the Chlorination Site

To determine the chlorination site of tryptophan by SnFDHal, an HPLC-based method was employed. The protocol achieved sufficient separation of 5-chlorotryptophan (5-Cl-Trp), 6-chlorotryptophan (6-Cl-Trp), and 7-chlorotryptophan (7-Cl-Trp), allowing identification of the halogenation product of SnFDHal by comparing the retention times to those of authentic standards. A gradient elution was performed using solvent A (acetonitrile) and solvent B (water containing 0.1% formic acid) under the following conditions: 0–5 min, B at 1%; 5–29 min, B at 25%; 29–35 min, B at 80%; 35–50 min, B at 5%, with a flow rate of 1 mL/min. L-tryptophan, its chlorinated derivatives, and products from SnFDHal-catalyzed reactions were analyzed using this method at a detection wavelength of 230 nm.

### Optimization of *In Vitro* Halogenation Conditions

The enzymatic activity of SnFDHal was assessed across a pH range of 3.0 to 9.0 for up to 30 min at a constant reaction temperature of 35 °C. To determine the optimal reaction temperature, enzymatic activity was also evaluated at temperatures ranging from 20 °C to 50 °C. All reactions were quenched and analyzed as described above. Each experiment was performed in triplicate to ensure reproducibility of the results.

### Determination of Melting Temperature (T_m_) for SnFDHal

The SnFDHal was freshly prepped and diluted to 5.5 µM in 100 mM phosphate buffer containing 10 mM NaCl at the desired pH. Invitrogen SYPRO Orange (Thermo Fisher) was added to a final working concentration of 5x. Fluorescence intensity was measured at 570 nM using an ISS PC1 spectrofluorometer over a temperature range of 20–75 °C. The data were normalized, plotted, and fitted to a Boltzmann sigmoid equation using GraphPad Prism version 10.0.3.

### Kinetic Analysis of SnFDHal

To measure the steady-state kinetic parameters (V_max_, *K*_*m*_, *k*_*cat*_ and catalytic efficiency) of SnFDHal for the chlorination of tryptophan and its derivatives, a series of 100 µL reactions was prepared in halogenation buffer containing varying substrate concentrations (20–1000 µM) in 100 mM phosphate buffer (pH 6.0). Reactions were incubated at 35 °C for 30 min, then quenched by adding 100 µL of methanol. Product formation was quantified by HPLC using the protocol described above. All reactions were performed in triplicate, and kinetic parameters were determined by nonlinear regression fitting to the Michaelis–Menten equation using Prism version 10.2.2 (GraphPad Software, La Jolla, CA, USA).

### Isolation of the Halogenated Product for NMR Analysis

To isolate the halogenated product of L-tryptophan (P1) by SnFDHal for structure elucidation, expression of pET28a-SnFDHal was performed as described in the purification section. Following an 18-hour post-induction period, the culture was centrifuged at 13,000 rpm for 8 min, and the resulting pellet was resuspended in 100 mM phosphate buffer (pH 6.0) supplemented with 1 mM substrate and glucose. The suspension was incubated for an additional 48 h at 28 °C. The culture broth was then centrifuged at 4,000 rpm for 10 min, and the supernatant was loaded onto a Diaion^®^ HP-20 column, followed by elution with a methanol–water mixture (1:1, v/v). The product-containing fractions were concentrated using a rotary evaporator and further purified by reverse-phase HPLC using a methanol–water gradient (10–50%, 0–17 min; 50–95%, 17–22 min) containing 0.1% formic acid (v/v). The purified product was dissolved in deuterated methanol (CD_3_OD) for NMR analysis. Spectra were recorded on a Bruker Avance III HD Ascend-500 NMR instrument (500 MHz for ^1^H NMR). Chemical shift (*δ*) values are presented in parts per million (ppm) and coupling constants (*J* values) in hertz (Hz).

### Molecular Modelling of SnFDHal

AlphaFold2 was used to generate a model of SnFDHal with F49 loop in a position that is comparable to PyrH in the substrate bound state. To dock the L-Trp substrate into the active site of the SnFDHal model, AutoDock Tools (version 1.5.7) was used to generate PDBQT files for L-Trp and SnFDHal, which were then used to run AutoDock Vina (version 4.2.6). The best Autodock solution placed L-Trp into the active site of SnFDHal with the C-5 site positioned for attacked by the conserved K75 and E359 catalytic residues. To place FAD and HClO within the FAD binding pocket of SnFDHal, we superimposed a structure of PrnA in complex with FAD and Cl^−^ (PDB ID: 2AQJ). The Cl^−^ ion was manually changed to HClO in MAESTRO (version 13.5.128), and the placement of FAD and HClO was energy minimized by applying the OPLS_2005 force field. The final model was visualized in ChimeraX (version 1.9). To position L-Trp into the active site of SnFDHal for attack at the C-5, C-6, or C-7 positions, we superimposed L-Trp-halogenase complex structures with known specificities (PDB Codes: 2WET for C5, 6UL2 for C6, and 2AQJ for C7).

## Results

### Discovery of a Putative Halogenase Gene From *S. noursei *NRRL B-1714

**Fig. 1:**
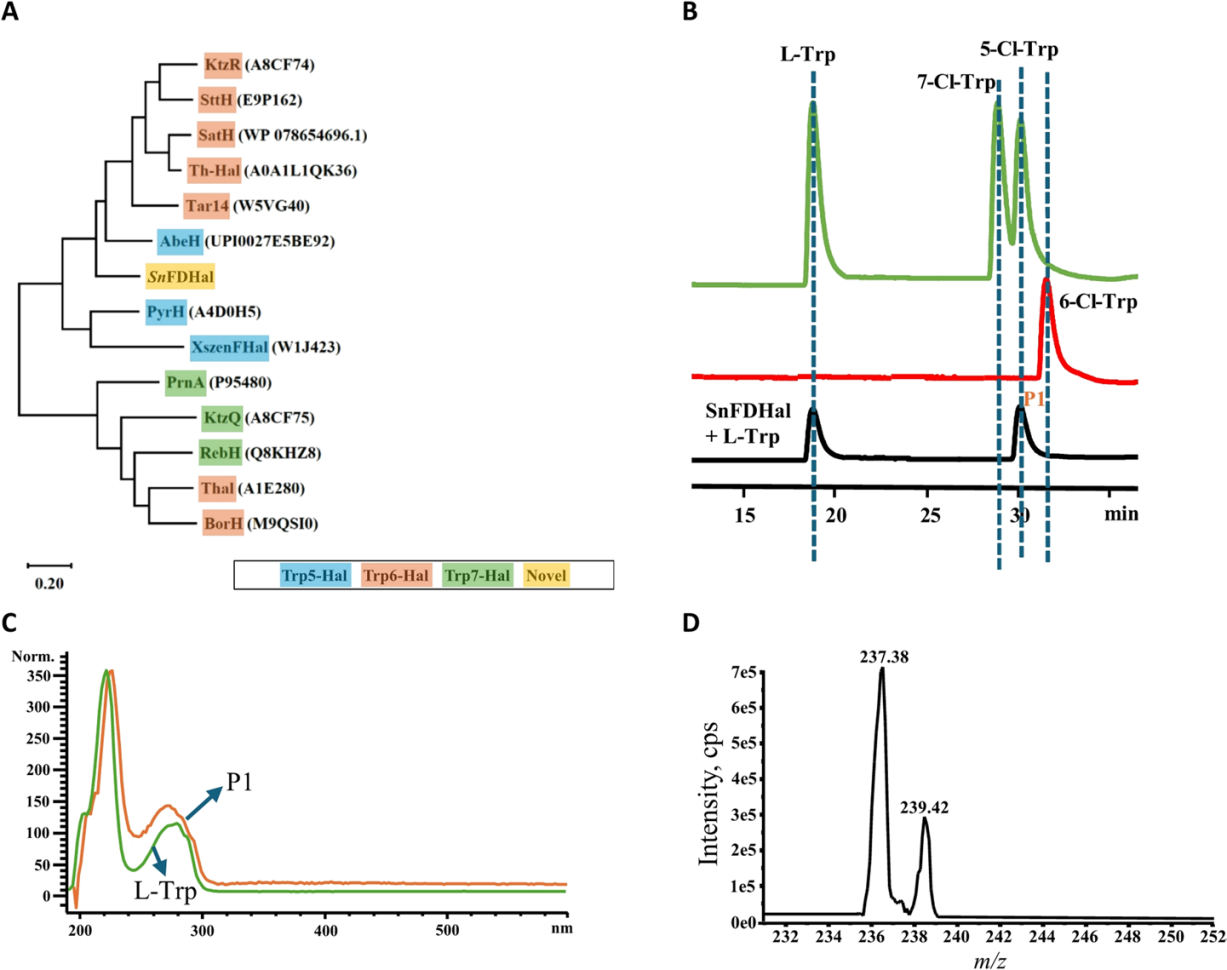
Analysis and functional characterization of SnFDHal. (**A**) Phylogenetic tree of flavin-dependent tryptophan halogenases including SnFDHal; (**B**) HPLC chromatograms of L-Trp, 5-Cl-Trp, 6-Cl-Trp, 7-Cl-Trp and the *in vitro* reaction mixture catalyzed by SnFDHal; (**C**) UV spectrum comparison of Trp and P1; (**D**) ESI-MS (-) spectrum for P1 (*m/z* 237.38 and 239.42)

The whole genome sequence of *S. noursei* NRRL B-1714 was annotated by the RAST annotation server [26], which revealed the presence of a putative flavin-dependent halogenase. Although halogenase genes are sometimes located within biosynthetic gene clusters, this particular gene seems to exist as an independent orphan gene. A BLAST search was conducted in the NCBI database which showed that the discovered halogenase has high sequence similarity with other reported tryptophan flavin-dependent halogenases (FDHs). We aligned this putative halogenase, named SnFDHal, with other tryptophan halogenases exhibiting varying regioselectivity, including Trp 5-, Trp 6-, and Trp 7-halogenases, using MUSCLE alignment (Fig. 1A). The analysis revealed that SnFDHal clustered closely with other Trp-5 halogenases, such as PyrH, *Xszen*FHal, and AbeH. This bioinformatic analysis suggests that this new halogenase may catalyze halogenation at the C-5 position of tryptophan or indole derivatives. However, experimental validation was necessary to confirm its regioselectivity.

### Functional Characterization of SnFDHal

To experimentally determine the function of SnFDHal, a halogenation assay was performed using purified SnFDHal in the presence of L-tryptophan as the substrate and sodium chloride as the halogen source. HPLC analysis of the *in vitro* reaction revealed the appearance of a single product peak (P1) in the chromatogram (Fig. 1B). The UV spectrum of P1 was highly similar to that of the substrate, with a slight bathochromic shift of P1 relative to L-Trp, indicating that P1 is likely a chlorinated derivative (Fig. 1C). We further confirmed the C-5 regioselectivity of SnFDHal via HPLC and found that the retention time of P1 aligns with that of the 5-Cl-Trp standard (Fig. 1B). Additionally, mass spec analysis of P1 displayed two major peaks at m/z 237.38 and 239.42, with an intensity ratio of 3:1, consistent with the expected isotopic pattern of a monochlorinated tryptophan (Fig. 1D). These results indicate SnFDHal catalyzes the chlorination of L-tryptophan at the C-5 position.

### Structure Identification of P1 Regioselectivity of SnFDHal by NMR Analysis

The chemical structure of P1 was confirmed by ^1^H NMR spectroscopy. Its ¹H NMR spectral data revealed the presence of one typical aromatic successive spin system with signals at δH 7.76 (d, J = 2.0 Hz, H-4), 7.10 (dd, J = 8.6, 2.0 Hz, H-6), and 7.35 (d, J = 8.6 Hz, H-7), as well as another aromatic singlet at δH 7.26 (s, H-2). The segment of –CH₂–CH– was also indicated by the clear signals at δH 3.85 (dd, J = 9.0, 4.2 Hz, H-11), 3.47 (dd, J = 15.2, 4.2 Hz, H-10a), and 3.14 (dd, J = 15.2, 9.0 Hz, H-10b). These data collectively confirmed the structure of SnFDHal P1 as 5-chlorotryptophan (SI Fig. 2).

### Molecular Modelling of SnFDHal

**Fig. 2:**
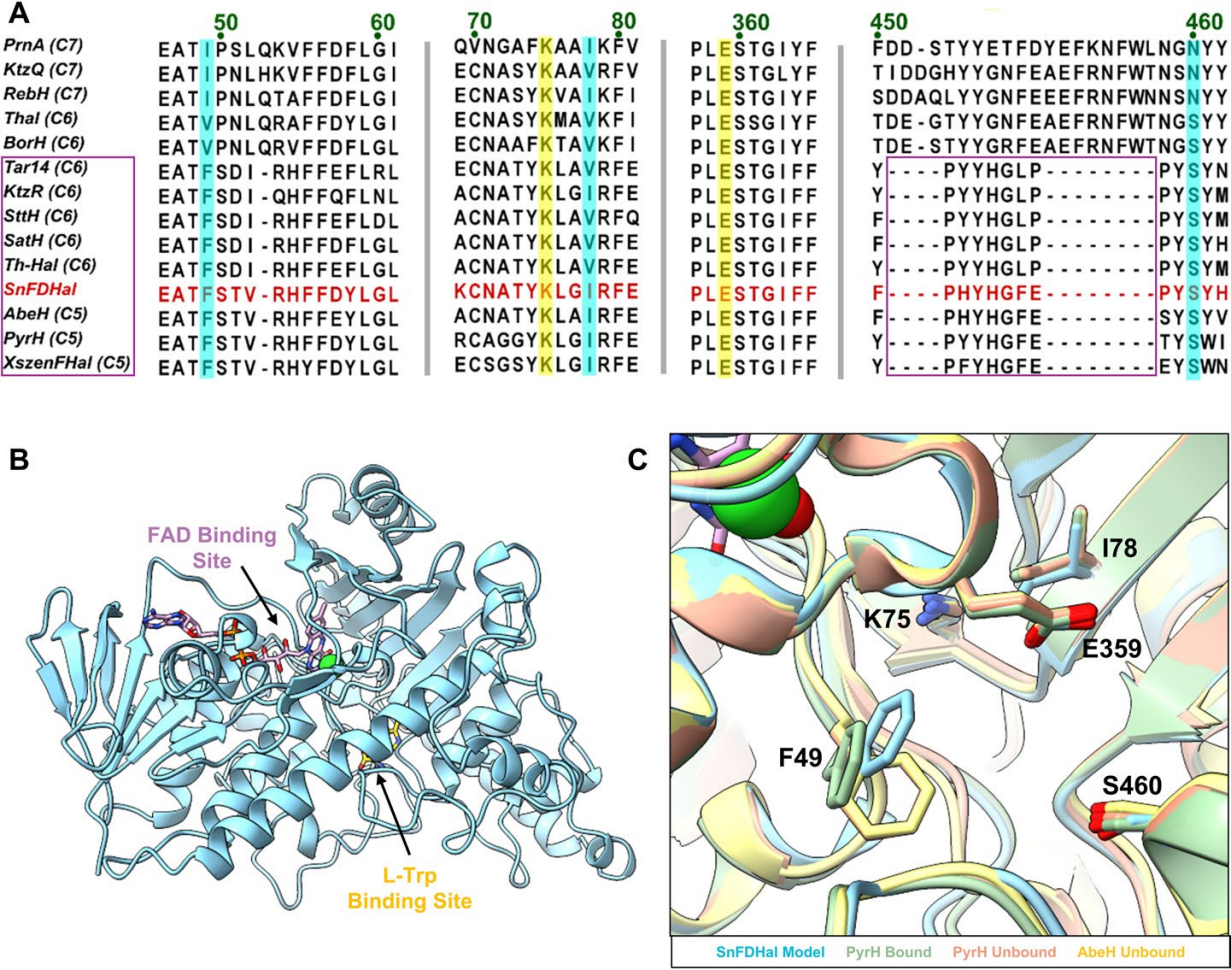
(**A**) Abbreviated multiple sequence alignment of Trp halogenases of known specificity. Catalytic residues are highlighted in yellow, non-conserved active site residues are highlighted in blue. Halogenases that contain a distal deletion are boxed in purple. The novel SnFDHal sequence is shown in red; (**B**) Model of SnFDHal with FAD (pink) and L-Trp (yellow) positioned into the FAD-binding site and the SnFDHal active site, respectively; (**C**) Superimposition of PyrH unbound (peach, PDB Code: 2WET chain A) and PyrH bound (green, PDB Code: 2WET chain B), and AbeH (yellow, PDB Code: 8FOV) onto SnFDHal model (blue)

A BLAST search of the NCBI database revealed that the new halogenase, SnFDHal, shares high sequence similarity with other reported flavin-dependent tryptophan halogenases (Fig. 2A, SI Fig. 4). SnFDHal was aligned with tryptophan halogenases of known regioselectivity, including Trp 5-, Trp 6-, and Trp 7-halogenases, and was found to cluster with those containing a distal deletion, such as XszenFHal, AbeH, PyrH, Tar14, KtzR, SttH, SatH, and Th-Hal (Fig. 2A, SI Fig. 4). The key catalytic residues K75 and E359, corresponding to positions K79 and E346 in PrnA, are 100% conserved among all known tryptophan halogenases. To identify additional residues potentially involved in substrate binding or regioselectivity, we generated a structural prediction of SnFDHal using AlphaFold2 (Fig. 2B). The resulting prediction was folded with high confidence, with an overall predicted template modeling (pTM) score of 0.93. Most residues had high or very high predicted local distance difference test (pLDDT) scores, except for the highly flexible FAD loop and the proximal insertion loop that are not near the L-Trp binding site (SI Fig. 5A). Collectively, the well folded prediction of this putative halogenase gene and the sequence similarity with other known Trp halogenases suggests that this gene likely encodes a novel Trp halogenase.

Based on the model, we identified three active site amino acids (F49, I78, and S460) of SnFDHal that differ across the known Trp halogenases which vary in specificity (Fig. 2C). Superimposition of the SnFDHal structural model with the published structures of other Trp 5-halogenases, PyrH and AbeH, revealed that the phenyl ring of F49 undergoes a conformational shift upon substrate binding, while the catalytic residues K75, E359, I78, and S460 remain in a conserved configuration. In the structure of PyrH bound to tryptophan, F49 moves away from the catalytic residues K75 and E359, creating space to accommodate the substrate (SI Fig. 5B). Thus, we modeled F49 in an outward conformation, mimicking the conformation of substrate binding (Fig. 2C). Interestingly, at the 49th position all Trp 5-halogenases have a bulkier phenylalanine at this position, while Trp 7-halogenases have a slightly smaller isoleucine side chain at this position (Fig. 2A). Trp 6-halogenases with a distal deletion possess a phenylalanine at position 49, similar to Trp 5-halogenases, whereas Trp 6-halogenases lacking the distal deletion typically have a valine at this position. This side chain preference at position 49 for each halogenase type suggests that the residue at this position may influence regioselectivity, a concept further supported by a recent study of the 6,7-dihalogenase SsDiHal [27]. Interestingly, engineering of the recently discovered SsDiHal demonstrated that subtle mutations to the three key sites we identified in SnFDHal (positions 49, 78, and 460 corresponding to positions 53, 83 and, 470 in SsDiHal) resulted in drastic improvements in the catalytic efficiency and regioselectivity for SsDiHal. The V53I or V53I/I83V *Ss*DiHal mutants exhibited a > 5-fold increase in catalytic efficiency relative to the wildtype, and a N470S mutant increased the enzyme’s catalytic efficiency on a similar scale while also changing the initial regioselectivity of the dihalogenase from a Trp 7-halogenase to a Trp 6-halogenase.

**Fig. 3:**
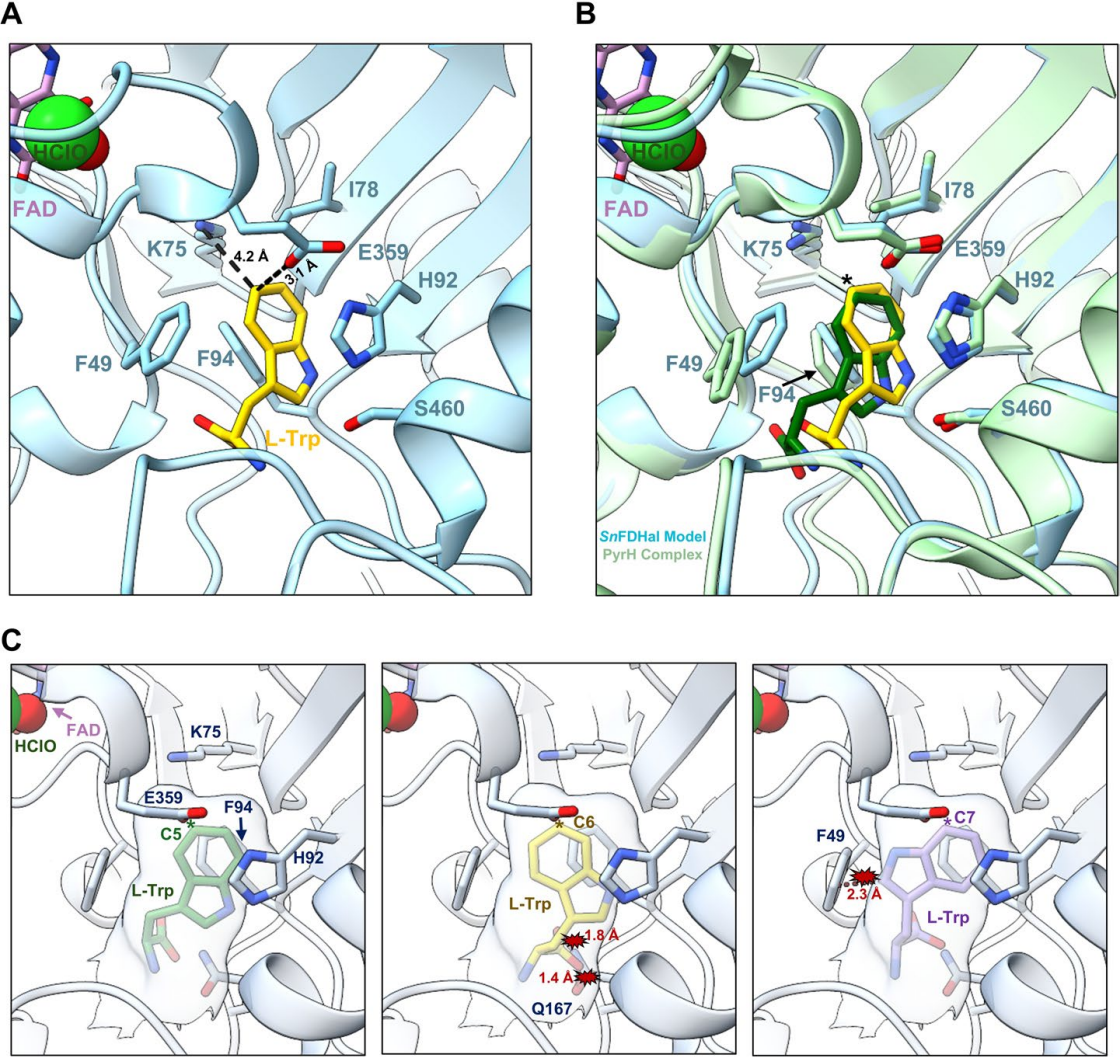
(**A**) Model of SnFDHal active site with L-Trp positioned for attack at the 5th carbon. Key residues are shown as sticks, and distances from the catalytic K75 and E359 to the C5 position of L-Trp residue are shown; (**B**) Superimposition of SnFDHal-Trp complex model onto the structure of PyrH bound to Trp (PDB Code: 2WET, chain B). The C5 halogenation site is indicated with an * in the figure; (**C**) L-Trp positioned for attack in the active site of SnFDHal at the C5 site (left), C6 site (middle), or C7 site (right). The L-Trp position was obtained by superimposing structures of halogenases with L-Trp bound (PDB Codes: 2WET for C5, 6UL2 for C6, and 2AQJ for C7)

To generate a model of SnFDHal bound to L-Trp, we used AutoDock Vina to dock the L-Trp substrate within the SnFDHal active site (Fig. 3A). Our final model shows L-Trp in a similar orientation to that observed in the PyrH complex structure, which is the only available structure of a C-5 halogenase in complex with Trp (Fig. 3B). In our model, the benzene portion of the L-Trp indole ring is sandwiched between the aromatic F94 and H92 side chains, forming π-π stacking interactions. Additionally, the C-5 position of L-Trp is positioned 3.1 Å and 4.2 Å away from the catalytic E359 and K75 residues respectively (Fig. 3A), a distance consistent with catalytic reaction. To better understand the C-5 specificity of SnFDHal we superimposed different Trp-halogenase complex structures where L-Trp is oriented for attack at either the C-6, or C-7 position onto our SnFDHal model and looked for any potential clashes when the L-Trp substrate is oriented for attack at each position (Fig. 3C). If L-Trp is positioned for attack at the C-6 position, the carbonyl group of L-Trp clashes with the neighboring Q187. Additionally, if L-Trp is oriented for attack at the C-7 position, the indole of L-Trp would clash with the bulky F49 side chain. Taken together, our structural analysis of other halogenases and the predicted structure of SnFDHal shows that the SnFDHal active site is comparable to the other known C-5 halogenases, PyrH and AbeH, and suggests that the presence of a bulkier F49 side chain within the SnFDHal active site supports orientation of the L-Trp substrate for attack at the C-5 position, which is consistent with the initial functional study results of this enzyme.

### Investigation of the Substrate Flexibility and Thermostability of SnFDHal and Optimization of the *In Vitro* Reaction Conditions

**Fig. 4:**
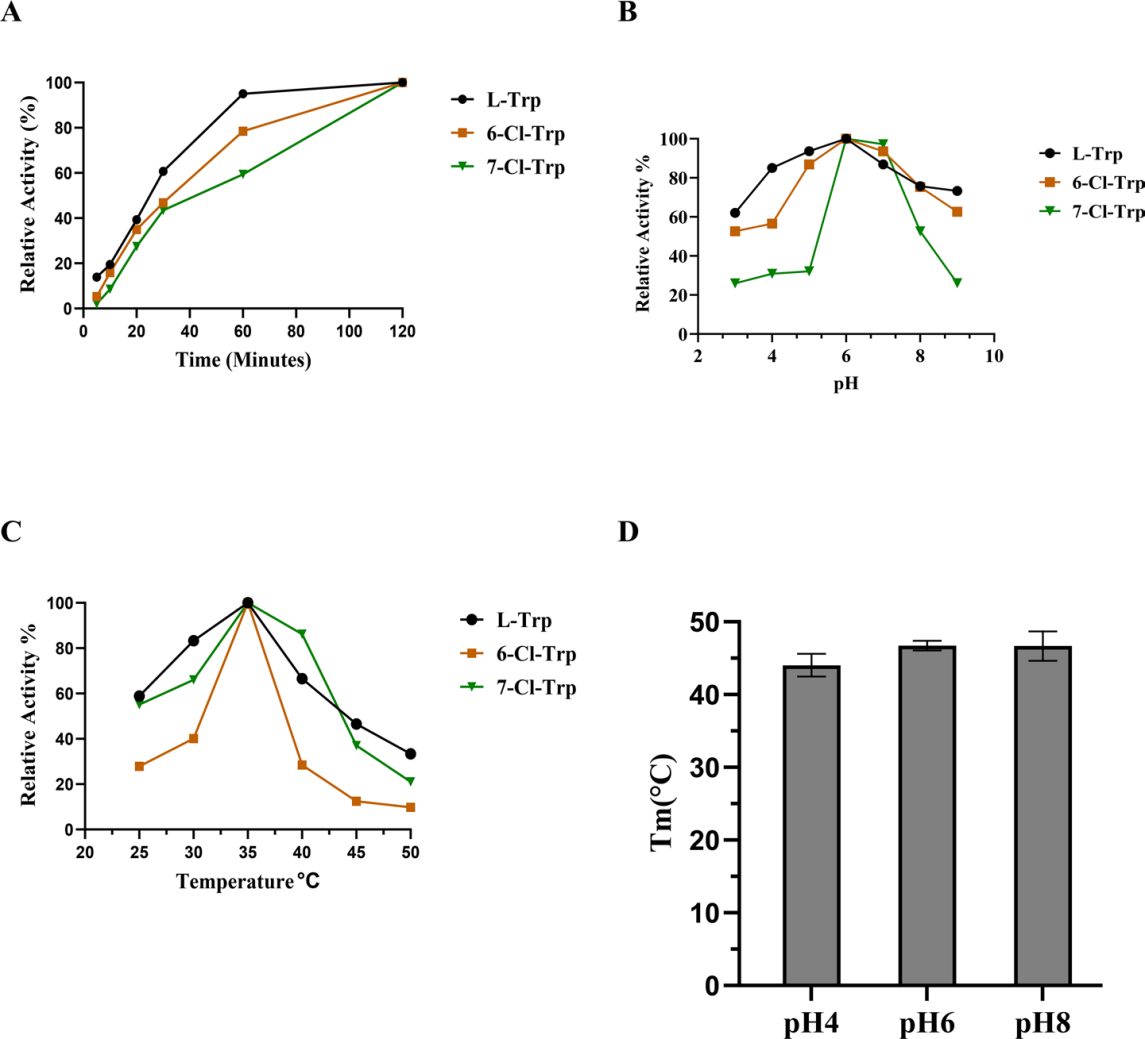
Thermostability of SnFDHal and optimization of its *in vitro* reaction conditions. (**A**) Time course analysis of the chlorination of Trp and their derivatives by SnFDHal; (**B**) Effect of pH on the chlorination activity of SnFDHal; (**C**) Effect of temperature on the chlorination activity of SnFDHal; (**D**) SnFDHal melting temperature at multiple pH levels

After confirming that SnFDHal can chlorinate L-tryptophan, we next investigated whether this halogenase could accept tryptophan derivatives as substrates. Indeed, we found that it was capable of further halogenating both 7-chlorotryptophan and 6-chlorotryptophan (SI Fig. 3A and B). We subsequently assessed the optimal pH, temperature range, and thermostability of SnFDHal. Time-dependent chlorination of L-tryptophan and their derivative was monitored over a period of 5 to 120 min, with maximum activity observed at 60 min for L-Trp, highlighting the catalytic efficiency of the enzyme (Fig. 4A). Additionally, SnFDHal demonstrated activity across a broad pH range of 5.0 to 8.0, with optimal activity at pH 6.0 (Fig. 4B). SnFDHal exhibited maximum activity between 30 °C and 40 °C, with a sharp decline in activity at temperatures above 40 °C (Fig. 4C). The melting temperature (T_m_) of SnFDHal was determined to be 46.7 ± 0.7 °C at pH 6.0 and 46.7 ± 2.0 °C at pH 8.0 (Fig. 4D). This melting temperature is comparable to those of some thermostable halogenases, such as BorH (48.0 °C) and Th-Hal (47.8 °C), both of which have reported T_m_ values 7–17 °C higher than other halogenases like PyrH, PrnA, KtzR and RebH [22, 28]. The broad pH tolerance and favorable thermostability of SnFDHal suggest that it is a stable, efficient, and fast-acting enzyme, offering potential as a greener, biocatalytic alternative to traditional chemical halogenation methods.

### Kinetic Studies on SnFDHal

The steady-state kinetic parameters for SnFDHal were determined as described in the Materials and Methods section. When L-tryptophan was used as the substrate, the enzyme exhibited a *K*_*m*_ of 0.6216 mM, a *k*_*cat*_ of 243.09 min⁻¹, and a catalytic efficiency (*k*_*cat*_/*K*_*m*_) of 391.07 min⁻¹ mM⁻¹ (Table 1). Notably, SnFDHal demonstrated higher catalytic efficiency for tryptophan than previously reported halogenases, including Thal-Hal at 30 °C (*k*_*cat*_/*K*_*m*_ = 350 min⁻¹ mM⁻¹), PyrH (160 min⁻¹ mM⁻¹), SttH (70 min⁻¹ mM⁻¹) [22], and Tar14 (35 min⁻¹ mM⁻¹) [29]. In addition to L-tryptophan, SnFDHal also accepted 6-chlorotryptophan and 7-chlorotryptophan as substrates, enabling the synthesis of dichlorinated tryptophan derivatives (SI Fig. 3A and B). For 6-chlorotryptophan, the *K*_*m*_, *k*_*cat*_, and *k*_*cat*_/*K*_*m*_ values were 0.945 mM, 91.27 min⁻¹, and 96.59 min⁻¹ mM⁻¹, respectively, while for 7-chlorotryptophan, the values were 0.1718 mM, 22.09 min⁻¹, and 128.59 min⁻¹ mM⁻¹ (Table 1). These results highlight the exceptional catalytic performance of SnFDHal with tryptophan and its halogenated derivatives, suggesting its potential versatility as a biocatalyst.

**Table 1:** Steady state kinetic parameters for SnFDHal-catalyzed chlorination of Trp, 6-Cl-Trp and 7-Cl-Trp. The values shown represent the mean of three replicates

Substrate	K_m_ (mM)	k_cat_ (Min^− 1^)	*k*_cat_/K_m_ (Min^− 1^mM^− 1^)
L-Trp	0.6216	243.09	391.07
6-Cl-Trp	0.9451	91.273	96.591
7-Cl-Trp	0.1718	22.092	128.58

## Discussion

In this study, we report the discovery and functional characterization of an efficient tryptophan 5-halogenase from *S. noursei* NRRL B-1714, identified through bioinformatic data mining. While flavin-dependent tryptophan halogenases targeting the C-6 and C-7 positions have been extensively studied, to date only few C-5 halogenases (PyrH, AbeH, and XszenFHal) have been reported [21, 30].

The majority of flavin-dependent halogenases exhibit maximum activity at moderate temperatures (25–45 °C) and within a neutral to slightly alkaline pH range (6.5–8.0). While some thermophilic halogenases, such as Th-Hal, demonstrate enhanced stability and activity at higher temperatures, most other halogenases are mesophilic, including PyrH and PrnA, which function optimally at lower temperatures [22]. The enzyme shows an optimal activity temperature of 35 °C and a pH optimum of 6.0, consistent with the activity profiles of other known halogenases. A time-course study of SnFDHal revealed rapid enzymatic activity, with product formation detectable within 5 min. This swift catalysis distinguishes SnFDHal from previously characterized halogenases, which typically require longer reaction times. For example, the most efficient reported halogenase, Th-Hal, achieves 69% conversion of 0.5 mM tryptophan to halogenated products in over 30 min, whereas SnFDHal completes over 95% conversion of 1 mM tryptophan into product within 60 min [22]. Another halogenase, PyrH, chlorinates 58% of 0.5 mM L-Trp in 25 h, highlighting its significantly slower turnover compared to SnFDHal [30].

Thermostability is a crucial property for enzymes intended for industrial applications, as it allows them to function efficiently at elevated temperatures, enhancing reaction rates and improving substrate solubility [31, 32]. SnFDHal functions over a broad pH range and exhibits a melting temperature of 46.7 °C at both pH 6.0 and pH 8.0, comparable to the thermophilic halogenases Th-Hal (47.8 °C) and BorH (48.0 °C) [22, 28] and significantly higher than those of mesophilic halogenases, which typically display melting temperatures below 40 °C [18, 22].

The enhanced thermostability of SnFDHal makes it a promising candidate for industrial applications, unlike mesophilic halogenases, which generally lack stability and catalytic efficiency at elevated temperatures. A broader analysis of long-term stability of SnFDHal under different storage and reaction conditions will be an important direction for future work. Steady-state kinetic analysis of SnFDHal for the chlorination of L-tryptophan and its derivatives revealed high catalytic efficiency, particularly for L-tryptophan, with a *k*_cat_ of 243.09 min⁻¹ and a *k*_cat_/*K*_m_ of 391.07 min⁻¹ mM⁻¹. These values are higher than those reported for other FDHs such as Th-Hal, Tar14, SttH, and RebH. The combination of high catalytic efficiency and favorable thermostability highlights SnFDHal’s potential as a potent biocatalyst for synthetic and industrial halogenation applications.

## Conclusion

In this work, we discovered a novel tryptophan halogenase, SnFDHal, from *S. noursei* NRRL B-1714 through bioinformatic mining. We demonstrated that this enzyme catalyzes the regioselective chlorination of L-tryptophan at the C-5 position. Structural analysis of SnFDHal, compared to other C-5 halogenases, revealed that a bulky phenylalanine residue at position 49 within the active site promotes substrate orientation, favoring halogenation at the C-5 position. Additionally, SnFDHal accepts both 6-chlorotryptophan and 7-chlorotryptophan as substrates, enabling the synthesis of di-chlorotryptophan derivatives. Thermal stability assays showed that SnFDHal possesses melting temperatures (T_m_) 46.7 °C at pH 6.0 and pH 8.0, comparable to thermophilic halogenases such as Th-Hal (47.8 °C) and BorH (48.0 °C), and significantly higher than mesophilic halogenases (T_m_ < 40 °C). Steady-state kinetic analyses further demonstrated that SnFDHal exhibits higher catalytic efficiency for L-tryptophan and its derivatives than previously reported flavin-dependent halogenases (FDHs). The combination of high catalytic efficiency and favorable thermostability makes SnFDHal a valuable addition to the family of FDHs, with promising potential for application in synthetic and biocatalytic processes within the agrochemical and pharmaceutical industries.

## Electronic supplementary material

Below is the link to the electronic supplementary material.

Supplementary Material 1 (DOCX 6.04 MB)

## Data Availability

The data are available in the supplementary file that supports the findings of this study.
