# Supplementary material for: Discovery and Functional Characterization of SnFDHal, an Efficient Tryptophan 5-Halogenase from Streptomyces noursei
Source: Appl Biochem Biotechnol. 2025 Nov 8;198(1):400–14. doi: 10.1007/s12010-025-05449-0 (PMC12894115; doi:10.1007/s12010-025-05449-0)
Supplement: Supplementary file 1 — Supplementary Material 1 (DOCX 6.04 MB) [file 12010_2025_5449_MOESM1_ESM.docx]

**Supplementary Information**

***for***

**Discovery and Functional Characterization of SnFDHal, an Efficient Tryptophan 5-Halogenase from *Streptomyces noursei***

Hassan Sher^1^, Haley A. Hardtke^2^, Mark D. Gold^3^, Sean J. Johnson^3^, Y. Jessie Zhang^2^, Jixun Zhan^1,*^

^1^ Department of Biological Engineering, Utah State University, Logan, UT 84322, United States

^2^ Department of Molecular Biosciences, The University of Texas at Austin, Austin, TX 78712, United States

^3^ Department of Chemistry and Biochemistry, Utah State University, Logan, Utah 84322, United States

* Corresponding author: J. Zhan ([jixun.zhan@usu.edu](mailto:jixun.zhan@usu.edu))

**B**

**A**

**SI Fig. 1**. (A) Digestion check of constructed pET28a-SnFDHal with NdeI and Xho1. (B) SDS-PAGE analysis of purified SnFDHal.


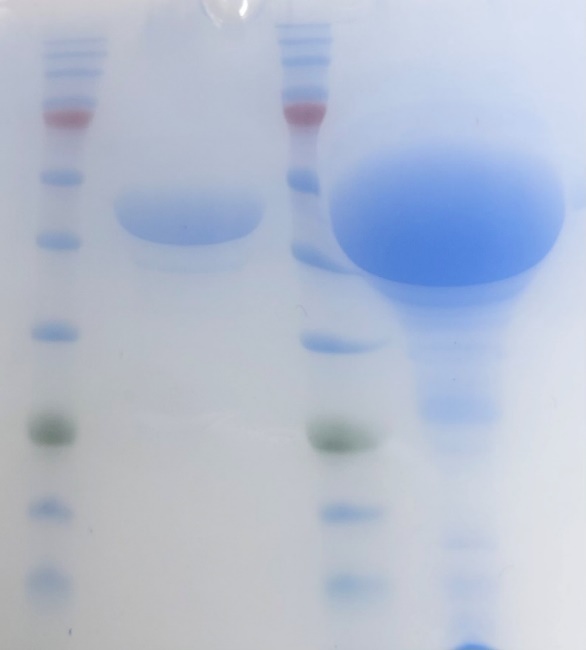

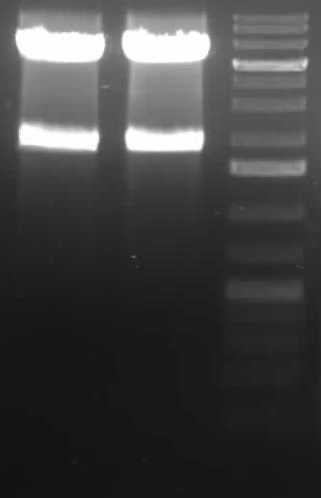


**5000**

**1500**

**500**

**pET28a**

***SnFDHal Insert***

**bp**

**SnFDHal**

**75**

**48**

**25**

**kDa**

**SI Fig. 2**. ^1^H NMR (500 MHz) spectrum of P1 of SnFDHal in CD_3_OD.

**SI Fig. 3.** (A) HPLC chromatograms showing the formation of new peak in *in vitro* reaction of SnFDHal with 6-chlorotryptophan (6-Cl-Trp) and 7-chlorotryptophan (7-Cl-Trp) as substrates. The new peak of both substrates, appearing at longer retention times than the substrates, suggest the formation of putative di-chlorinated tryptophan products. The retention times of both putative di-chlorinated tryptophan products is same with the retention time of the confirmed 6,7-di-chlorinated tryptophan (6,7-Cl-Trp) produced by *Sn*DiHal. (B) UV absorption spectra of 6-Cl-Trp, 7-Cl-Trp, and the enzymatic di-chlorinated product from SnFDHal, compared with the 6,7-di-Cl-Trp product from *Ss*DiHal.

**B**

**A**

**SI Fig. 4.** Multiple sequence alignment of tryptophan halogenases with known substrate specificities. Catalytic residues are highlighted in yellow, while non-conserved active site residues are shown in blue. The proximal deletion region is indicated by a red box, and the distal deletion region is marked with a purple box. The sequence of the novel enzyme **SnFDHal** is displayed in red.

**B**

**A**

**SI Fig. 5. (**A) Alphafold2 model of SnFDHal colored by pLDDt score. (B) Superimposition of PyrH unbound (peach, PDB Code: 2WET chain A) and PyrH bound (green, PDB Code: 2WET chain B) to the L-Trp substrate (dark green).

ATGCTTTCCAGCGTAGTTATTGTCGGCGGCGGCACATCGGGCTGGATGACCGCCGCGTATTTGCGGGCCGCGCTCGGTACGAGCGTCAATGTCACCGTCATCGAGTCCAAGCGGATCAAAACGATCGGCGTCGGCGAGGCCACGTTCTCCACCGTGCGCCACTTCTTCGACTACCTCGGCCTCTCGGAGAACGACTGGATGCCGAAGTGCAACGCCACCTACAAGCTCGGCATCCGCTTCGAGAACTGGCGCGCCAAGGGCCACTACTTCTACCACCCCTTCGAGCGGCTGCGGGTCGTCGACGGCTTCCCGCTCACCGAGTGGTGGCTGAACAAGAAGCCCAGCGACCGCTTCGACCAGGACGTCTTCCTGATGTCCGAGATCTGCGACACCATGCGCTCGCCGCGCTACCTGGACGGCACCCTCTTCGAGCAGGACTTCGTCGAGCACGGCGGCTCCATGGACCCGGAGCGCTCCACCCTCTCCGAACAGGCCACCCAGTTCCCCTACGCCTACCAGTTCGACGCGTCGCTGCTCGCCGACTTCCTCACCGAGTACGCCACCACGCGCGGTGCCCGGCACATCGAGGACGACGTGGTGGAGGTCGTCCGCGACGAGCGCGGCTGGATCAGCCATCTCAAGACCCGCGAACACGGCGAGCTGGCCGGCGACCTGTTCGTCGACTGCACCGGCTTCGCCGGCCTGCTGCTCAACAAGACGCTGGGCGAACCGTTCGTCTCGTACCAGAACACCCTGCCCAACGACAGCGCGGTGGCGCTGCGCGTCCCGCACGACGCCGAGCGCACCCGGCTGCGCCCGTGCACCACCGCCACCGCGCAGGAGGCCGGCTGGATCTGGACGATCCCGCTCTTCGAGCGCATCGGCACCGGCTACGTCTACGCCAGCGACTACACCACCCCCGAAGAGGCCGAGCGCACCCTGCGCGAATTCGTCGGCCCGCAGGCCGCGGACGTCGAGGCCAATCACATCCGCATGCGGATCGGCCGCAGCCGGCACTCCTGGGTCAACAACTGCGTGGCCATCGGCCTTTCCAGCGGATTCGTCGAGCCGCTGGAATCGACCGGCATCTTCTTCATCCAGCAGGGCATCGAGGAACTCGTCAAGCATTTCCCGGACGCGAAGTGGGACCCGAAGCTCCGCGATTCCTACAACCGCGTCGTGGCCAACACCATGGACGGCGTACGGGAATTCCTGGTGCTGCACTACCGCACCGCGGCCCGCAACGACAACGCCTATTGGCGGGACGCCAAGACCCGCGAACTCCCCGACGGGCTGGCCGCCCGCCTGGAGGCGTGGCAGTCCAAGCTGCCCACCGAGGAGACGGTCTTCCCGCACTACCACGGGTTCGAGCCCTACTCCTACCACGCGATGCTGCTCGGCCTCGGCGGCCTCGACGTCAAGCCCGCGCCCGTCCTCGCGCACATGGACGACTCGCGCGCCGCGCAGGAGATCCAGCGGCTCAAGGACCAGGCCCGCGACATCGCCAAGCGGCTGCCCAGCCAGTACGAGTACCTCGCCCAGATGCACTGA

MLSSVVIVGGGTSGWMTAAYLRAALGTSVNVTVIESKRIKTIGVGEATFSTVRHFFDYLGLSENDWMPKCNATYKLGIRFENWRAKGHYFYHPFERLRVVDGFPLTEWWLNKKPSDRFDQDVFLMSEICDTMRSPRYLDGTLFEQDFVEHGGSMDPERSTLSEQATQFPYAYQFDASLLADFLTEYATTRGARHIEDDVVEVVRDERGWISHLKTREHGELAGDLFVDCTGFAGLLLNKTLGEPFVSYQNTLPNDSAVALRVPHDAERTRLRPCTTATAQEAGWIWTIPLFERIGTGYVYASDYTTPEEAERTLREFVGPQAADVEANHIRMRIGRSRHSWVNNCVAIGLSSGFVEPLESTGIFFIQQGIEELVKHFPDAKWDPKLRDSYNRVVANTMDGVREFLVLHYRTAARNDNAYWRDAKTRELPDGLAARLEAWQSKLPTEETVFPHYHGFEPYSYHAMLLGLGGLDVKPAPVLAHMDDSRAAQEIQRLKDQARDIAKRLPSQYEYLAQMH

**A**

**SI Table 1.** (A) Amino acid sequences of the recombinant SnFDHal.  **(B)** DNA sequences of SnFDHal.

**B**

**SI Table 2.** List of oligos used in this study for amplification of SnFDHal*.*

| **Oligo Name** | **Sequence** |
| --- | --- |
| ***Sn*FDHal-Nde1-F** | cccCATATGCTTTCCAGCGTAGTTATTGTCGGCG |
| ***Sn*FDHal-EcoR1-R** | ccgGAATTCTCAGTGCATCTGGGCGAGG |
